# 5-(4-Bromo­phen­yl)-3-(4-fluoro­phen­yl)-1-phenyl-4,5-dihydro-1*H*-pyrazole

**DOI:** 10.1107/S160053681203454X

**Published:** 2012-08-11

**Authors:** Hoong-Kun Fun, Tze Shyang Chia, M. Sapnakumari, B. Narayana, B. K. Sarojini

**Affiliations:** a X-ray Crystallography Unit, School of Physics, Universiti Sains Malaysia, 11800 USM, Penang, Malaysia; b Department of Studies in Chemistry, Mangalore University, Mangalagangotri 574 199, India; c Department of Chemistry, P. A. College of Engineering, Nadupadavu, Mangalore 574 153, India

## Abstract

In the title compound, C_21_H_16_BrFN_2_, the fluoro-substituted benzene ring is disordered over two orientations about the C—F bond and the C—C bond between the benzene and pyrazole groups with a site-occupancy ratio of 0.516 (8):0.484 (8). The central pyrazole ring [maximum deviation = 0.035 (3) Å] makes dihedral angles of 22.4 (2), 11.0 (2), 77.19 (16) and 7.44 (17)° with the two disorder components of the benzene ring, the bromo-substituted benzene ring and the phenyl ring, respectively. In the crystal, mol­ecules are linked into a layer parallel to the *bc* plane through C—H⋯π inter­actions.

## Related literature

For background to pyrazoline derivatives, see: Fun *et al.* (2010 ▶); Samshuddin *et al.* (2010 ▶, 2011 ▶). For a related structure, see: Samshuddin *et al.* (2010 ▶). For the stability of the temperature controller used in the data collection, see: Cosier & Glazer (1986 ▶).
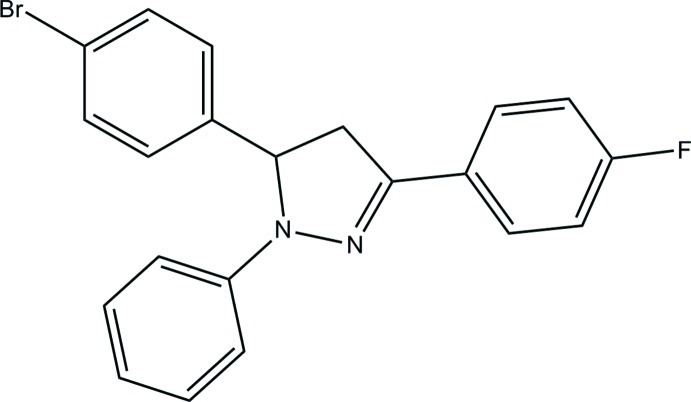


## Experimental

### 

#### Crystal data

C_21_H_16_BrFN_2_
*M* *_r_* = 395.27
Monoclinic, 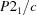

*a* = 20.5345 (5) Å
*b* = 5.2689 (1) Å
*c* = 16.1929 (5) Å
β = 104.443 (2)°
*V* = 1696.61 (7) Å^3^
*Z* = 4
Mo *K*α radiation
μ = 2.44 mm^−1^
*T* = 100 K
0.25 × 0.13 × 0.09 mm

#### Data collection

Bruker SMART APEXII CCD area-detector diffractometer
Absorption correction: multi-scan (*SADABS*; Bruker, 2009 ▶) *T*_min_ = 0.583, *T*_max_ = 0.818
16716 measured reflections
4974 independent reflections
3761 reflections with *I* > 2σ(*I*)
*R*_int_ = 0.048

#### Refinement

*R*[*F* ^2^ > 2σ(*F* ^2^)] = 0.051
*wR*(*F* ^2^) = 0.113
*S* = 1.01
4974 reflections
263 parameters
130 restraints
H-atom parameters constrained
Δρ_max_ = 1.26 e Å^−3^
Δρ_min_ = −0.99 e Å^−3^

### 

Data collection: *APEX2* (Bruker, 2009 ▶); cell refinement: *SAINT* (Bruker, 2009 ▶); data reduction: *SAINT*; program(s) used to solve structure: *SHELXTL* (Sheldrick, 2008 ▶); program(s) used to refine structure: *SHELXTL*; molecular graphics: *SHELXTL*; software used to prepare material for publication: *SHELXTL* and *PLATON* (Spek, 2009 ▶).

## Supplementary Material

Crystal structure: contains datablock(s) global, I. DOI: 10.1107/S160053681203454X/is5174sup1.cif

Structure factors: contains datablock(s) I. DOI: 10.1107/S160053681203454X/is5174Isup2.hkl

Supplementary material file. DOI: 10.1107/S160053681203454X/is5174Isup3.cml

Additional supplementary materials:  crystallographic information; 3D view; checkCIF report

## Figures and Tables

**Table 1:** Hydrogen-bond geometry (Å, °) *Cg*1 is the centroid of the C10–C15 ring.

*D*—H⋯*A*	*D*—H	H⋯*A*	*D*⋯*A*	*D*—H⋯*A*
C7—H7*A*⋯*Cg*1^i^	1.00	2.60	3.522 (3)	153
C17—H17*A*⋯*Cg*1^ii^	0.95	2.99	3.752 (6)	138

Symmetry codes: (i) 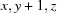; (ii) 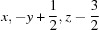.

**Fig. 1.**
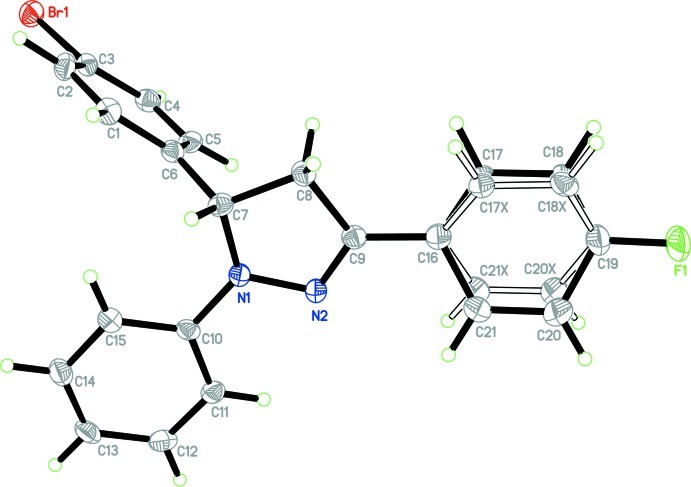
The molecular structure of the title compound with atom labels with 50% probability displacement ellipsoids. The minor component of disorder is indicated by the open bonds.

## supplementary crystallographic information

### Comment

In continuation of our work on synthesis of pyrazoline derivatives (Fun *et al.*, 2010; Samshuddin *et al.*, 2010, 2011), the title compound was prepared and its crystal structure is now reported.

The asymmetric unit of the title compound is shown in Fig. 1. The fluoro-substituted benzene ring is disordered over two positions (C16–C21 and C16/C17X/C18X/C19/C20X/C21X) rotated about the C9—C16···C19—F1 axis with a site-occupancy ratio of 0.516 (8):0.484 (8). The central pyrazole ring [N1/N2/C7–C9; maximum deviation = 0.035 (3) Å at atom C7] makes dihedral angles of 77.19 (16), 7.44 (17), 22.4 (2) and 11.0 (2)° with the C1–C6 (A), C10–C15 (B), C16–C21 (C) and C16/C17X/C18X/C19/C20X/C21X (D) benzene rings, respectively. The dihedral angles between the benzene rings are A/B = 83.25 (15)°, A/C = 87.1 (2)°, A/D = 67.2 (2)°, B/C = 22.6 (2)° and B/D = 16.1 (2)°. The bond lengths and angles are comparable to those found in a related structure (Samshuddin *et al.*, 2010). In the crystal, molecules are linked into a layer parallel to (100) through intermolecular C—H···π interactions (Table 1), involving *Cg*1 which is the centroid of the C10–C15 ring.

### Experimental

A mixture of (2*E*)-3-(4-bromophenyl)-1-(4-fluorophenyl)prop-2-en-1-one (3.05 g, 0.01 mol) and phenyl hydrazine (0.98 ml, 0.01 mol) in 50 ml of glacial acetic acid was refluxed for 6 h. The reaction mixture was cooled and poured into 50 ml ice-cold water. The precipitate was collected by filtration and purified by recrystallization from toluene. Orange blocks were grown from ethanol by slow evaporation method (m.p. 397–399 K).

### Refinement

All H atoms were positioned geometrically (C—H = 0.95, 0.99 and 1.00 Å) and refined using a riding model with *U*_iso_(H) = 1.2*U*_eq_(C). The fluoro-substituted benzene ring is statistically disordered over two conformations with a site-occupancy ratio of 0.516 (8):0.484 (8). Similarity (SAME), similar-ADP (SIMU) and FLAT restraints were used for the major and minor components of disordered fluoro-substituted benzene ring (C16–C21 and C16/C17X/C18X/C19/C20X/C21X). The highest peak is located at 0.31 Å from atom C17, whereas the deepest hole is located at 0.34 Å from atom C21.

### Crystal data

**Table:**

C_21_H_16_BrFN_2_	*F*(000) = 800
*M**_r_* = 395.27	*D*_x_ = 1.547 Mg m^−^^3^
Monoclinic, *P*2_1_/*c*	Mo *K*α radiation, λ = 0.71073 Å
Hall symbol: -P 2ybc	Cell parameters from 4161 reflections
*a* = 20.5345 (5) Å	θ = 2.9–30.0°
*b* = 5.2689 (1) Å	µ = 2.44 mm^−^^1^
*c* = 16.1929 (5) Å	*T* = 100 K
β = 104.443 (2)°	Block, orange
*V* = 1696.61 (7) Å^3^	0.25 × 0.13 × 0.09 mm
*Z* = 4	

### Data collection

**Table:**

Bruker SMART APEXII CCD area-detector diffractometer	4974 independent reflections
Radiation source: fine-focus sealed tube	3761 reflections with *I* > 2σ(*I*)
Graphite monochromator	*R*_int_ = 0.048
φ and ω scans	θ_max_ = 30.2°, θ_min_ = 2.1°
Absorption correction: multi-scan (*SADABS*; Bruker, 2009)	*h* = −29→28
*T*_min_ = 0.583, *T*_max_ = 0.818	*k* = −7→7
16716 measured reflections	*l* = −20→22

### Refinement

**Table:**

Refinement on *F*^2^	Primary atom site location: structure-invariant direct methods
Least-squares matrix: full	Secondary atom site location: difference Fourier map
*R*[*F*^2^ > 2σ(*F*^2^)] = 0.051	Hydrogen site location: inferred from neighbouring sites
*wR*(*F*^2^) = 0.113	H-atom parameters constrained
*S* = 1.01	*w* = 1/[σ^2^(*F*_o_^2^) + (0.0403*P*)^2^ + 3.9007*P*] where *P* = (*F*_o_^2^ + 2*F*_c_^2^)/3
4974 reflections	(Δ/σ)_max_ = 0.001
263 parameters	Δρ_max_ = 1.26 e Å^−^^3^
130 restraints	Δρ_min_ = −0.99 e Å^−^^3^

### Special details

**Table:**

Experimental. The crystal was placed in the cold stream of an Oxford Cryosystems Cobra open-flow nitrogen cryostat (Cosier & Glazer, 1986) operating at 100.0 (1) K.
Geometry. All e.s.d.'s (except the e.s.d. in the dihedral angle between two l.s. planes) are estimated using the full covariance matrix. The cell e.s.d.'s are taken into account individually in the estimation of e.s.d.'s in distances, angles and torsion angles; correlations between e.s.d.'s in cell parameters are only used when they are defined by crystal symmetry. An approximate (isotropic) treatment of cell e.s.d.'s is used for estimating e.s.d.'s involving l.s. planes.
Refinement. Refinement of *F*^2^ against ALL reflections. The weighted *R*-factor *wR* and goodness of fit *S* are based on *F*^2^, conventional *R*-factors *R* are based on *F*, with *F* set to zero for negative *F*^2^. The threshold expression of *F*^2^ > σ(*F*^2^) is used only for calculating *R*-factors(gt) *etc*. and is not relevant to the choice of reflections for refinement. *R*-factors based on *F*^2^ are statistically about twice as large as those based on *F*, and *R*- factors based on ALL data will be even larger.

### Fractional atomic coordinates and isotropic or equivalent isotropic displacement parameters (Å^2^)

**Table:**

	*x*	*y*	*z*	*U*_iso_*/*U*_eq_	Occ. (<1)
Br1	0.438172 (16)	0.29092 (7)	0.31781 (2)	0.02527 (11)	
F1	1.03501 (11)	1.0361 (5)	0.31750 (17)	0.0482 (7)	
N1	0.76496 (13)	0.5313 (5)	0.52597 (18)	0.0205 (6)	
N2	0.82467 (12)	0.5535 (5)	0.50180 (17)	0.0189 (5)	
C1	0.58983 (16)	0.7495 (6)	0.4593 (2)	0.0198 (6)	
H1A	0.5930	0.8942	0.4951	0.024*	
C2	0.52691 (16)	0.6511 (6)	0.4199 (2)	0.0210 (7)	
H2A	0.4872	0.7281	0.4282	0.025*	
C3	0.52306 (15)	0.4394 (6)	0.3684 (2)	0.0194 (6)	
C4	0.58002 (16)	0.3298 (6)	0.3531 (2)	0.0212 (6)	
H4A	0.5765	0.1866	0.3166	0.025*	
C5	0.64254 (16)	0.4316 (6)	0.3919 (2)	0.0202 (6)	
H5A	0.6819	0.3593	0.3808	0.024*	
C6	0.64832 (15)	0.6383 (6)	0.4469 (2)	0.0175 (6)	
C7	0.71689 (15)	0.7394 (6)	0.4928 (2)	0.0184 (6)	
H7A	0.7125	0.8523	0.5408	0.022*	
C8	0.75404 (15)	0.8818 (6)	0.4346 (2)	0.0193 (6)	
H8A	0.7292	0.8694	0.3740	0.023*	
H8B	0.7606	1.0630	0.4508	0.023*	
C9	0.82035 (15)	0.7440 (6)	0.4509 (2)	0.0183 (6)	
C10	0.76131 (15)	0.3686 (6)	0.59269 (19)	0.0171 (6)	
C11	0.81265 (15)	0.1909 (6)	0.6237 (2)	0.0202 (6)	
H11A	0.8515	0.1880	0.6020	0.024*	
C12	0.80621 (16)	0.0197 (6)	0.6863 (2)	0.0232 (7)	
H12A	0.8408	−0.1014	0.7068	0.028*	
C13	0.75020 (17)	0.0222 (7)	0.7196 (2)	0.0237 (7)	
H13A	0.7461	−0.0966	0.7621	0.028*	
C14	0.70032 (16)	0.2001 (7)	0.6900 (2)	0.0238 (7)	
H14A	0.6620	0.2038	0.7129	0.029*	
C15	0.70535 (16)	0.3739 (6)	0.6272 (2)	0.0213 (6)	
H15A	0.6708	0.4958	0.6078	0.026*	
C16	0.87551 (16)	0.8212 (6)	0.4136 (2)	0.0228 (7)	
C19	0.98129 (18)	0.9645 (7)	0.3479 (2)	0.0333 (8)	
C17	0.8674 (3)	0.9700 (12)	0.3440 (4)	0.0143 (13)	0.516 (8)
H17A	0.8232	1.0218	0.3153	0.017*	0.516 (8)
C18	0.9209 (4)	1.0507 (15)	0.3126 (5)	0.0138 (14)	0.516 (8)
H18A	0.9139	1.1664	0.2662	0.017*	0.516 (8)
C20	0.9970 (4)	0.8198 (14)	0.4250 (5)	0.0348 (17)	0.516 (8)
H20A	1.0420	0.7748	0.4526	0.042*	0.516 (8)
C21	0.9429 (3)	0.7482 (13)	0.4575 (5)	0.0287 (16)	0.516 (8)
H21A	0.9505	0.6513	0.5085	0.034*	0.516 (8)
C17X	0.8755 (4)	1.0417 (15)	0.3726 (4)	0.0186 (15)	0.484 (8)
H17B	0.8387	1.1544	0.3684	0.022*	0.484 (8)
C18X	0.9274 (5)	1.1126 (19)	0.3360 (5)	0.0211 (17)	0.484 (8)
H18B	0.9243	1.2636	0.3031	0.025*	0.484 (8)
C20X	0.9841 (3)	0.7160 (14)	0.3859 (4)	0.0226 (14)	0.484 (8)
H20B	1.0210	0.6052	0.3878	0.027*	0.484 (8)
C21X	0.9309 (3)	0.6444 (14)	0.4195 (4)	0.0200 (14)	0.484 (8)
H21B	0.9307	0.4834	0.4458	0.024*	0.484 (8)

### Atomic displacement parameters (Å^2^)

**Table:**

	*U*^11^	*U*^22^	*U*^33^	*U*^12^	*U*^13^	*U*^23^
Br1	0.01981 (15)	0.03041 (18)	0.02509 (18)	−0.00327 (13)	0.00464 (12)	−0.00043 (15)
F1	0.0264 (11)	0.0682 (17)	0.0583 (16)	0.0129 (11)	0.0263 (11)	0.0328 (14)
N1	0.0165 (12)	0.0241 (14)	0.0221 (14)	0.0036 (10)	0.0071 (11)	0.0078 (11)
N2	0.0164 (11)	0.0212 (14)	0.0202 (14)	0.0005 (10)	0.0064 (10)	0.0037 (11)
C1	0.0255 (15)	0.0129 (16)	0.0224 (15)	0.0022 (11)	0.0084 (13)	−0.0011 (12)
C2	0.0204 (14)	0.0199 (16)	0.0258 (17)	0.0022 (11)	0.0114 (13)	0.0010 (13)
C3	0.0180 (13)	0.0225 (16)	0.0182 (15)	−0.0019 (11)	0.0051 (12)	0.0032 (13)
C4	0.0254 (15)	0.0202 (17)	0.0179 (15)	0.0027 (12)	0.0050 (12)	−0.0005 (12)
C5	0.0201 (14)	0.0223 (16)	0.0182 (15)	0.0072 (12)	0.0050 (12)	0.0003 (12)
C6	0.0192 (14)	0.0164 (14)	0.0174 (15)	0.0020 (11)	0.0057 (12)	0.0026 (11)
C7	0.0182 (13)	0.0170 (16)	0.0198 (15)	0.0027 (11)	0.0043 (12)	0.0004 (12)
C8	0.0207 (14)	0.0174 (14)	0.0209 (16)	0.0019 (11)	0.0073 (12)	0.0021 (12)
C9	0.0192 (13)	0.0164 (16)	0.0193 (15)	0.0014 (11)	0.0048 (12)	0.0010 (12)
C10	0.0183 (13)	0.0182 (14)	0.0132 (14)	−0.0042 (11)	0.0008 (11)	0.0002 (11)
C11	0.0168 (13)	0.0219 (15)	0.0203 (15)	−0.0022 (12)	0.0017 (12)	0.0010 (13)
C12	0.0214 (15)	0.0212 (16)	0.0233 (17)	−0.0021 (12)	−0.0015 (13)	0.0038 (13)
C13	0.0279 (16)	0.0258 (17)	0.0165 (16)	−0.0049 (13)	0.0039 (13)	0.0062 (13)
C14	0.0247 (15)	0.0282 (17)	0.0197 (16)	−0.0057 (13)	0.0082 (13)	0.0009 (14)
C15	0.0218 (14)	0.0231 (16)	0.0193 (16)	0.0006 (12)	0.0055 (13)	0.0012 (13)
C16	0.0207 (14)	0.0230 (16)	0.0251 (16)	0.0023 (12)	0.0066 (12)	0.0045 (13)
C19	0.0246 (16)	0.042 (2)	0.038 (2)	0.0051 (15)	0.0157 (15)	0.0160 (17)
C17	0.011 (2)	0.022 (3)	0.007 (3)	−0.002 (2)	−0.004 (2)	0.002 (2)
C18	0.015 (3)	0.020 (4)	0.005 (3)	−0.004 (2)	0.000 (3)	−0.001 (2)
C20	0.023 (3)	0.041 (4)	0.040 (4)	0.003 (3)	0.005 (3)	0.014 (3)
C21	0.026 (3)	0.033 (4)	0.028 (3)	0.003 (3)	0.006 (3)	0.012 (3)
C17X	0.018 (3)	0.018 (3)	0.019 (4)	0.001 (3)	0.004 (3)	0.000 (3)
C18X	0.026 (3)	0.022 (4)	0.013 (4)	−0.004 (3)	0.001 (3)	0.000 (3)
C20X	0.022 (3)	0.026 (3)	0.020 (3)	0.001 (3)	0.006 (2)	−0.005 (3)
C21X	0.027 (3)	0.020 (3)	0.014 (3)	−0.001 (2)	0.005 (2)	−0.005 (2)

### Geometric parameters (Å, º)

**Table:**

Br1—C3	1.900 (3)	C12—C13	1.386 (5)
F1—C19	1.368 (4)	C12—H12A	0.9500
N1—N2	1.382 (3)	C13—C14	1.383 (5)
N1—C10	1.395 (4)	C13—H13A	0.9500
N1—C7	1.484 (4)	C14—C15	1.391 (5)
N2—C9	1.288 (4)	C14—H14A	0.9500
C1—C2	1.391 (5)	C15—H15A	0.9500
C1—C6	1.395 (4)	C16—C17X	1.338 (9)
C1—H1A	0.9500	C16—C17	1.349 (8)
C2—C3	1.383 (5)	C16—C21	1.441 (7)
C2—H2A	0.9500	C16—C21X	1.454 (7)
C3—C4	1.381 (4)	C19—C18	1.310 (9)
C4—C5	1.389 (4)	C19—C18X	1.328 (11)
C4—H4A	0.9500	C19—C20	1.429 (8)
C5—C6	1.393 (4)	C19—C20X	1.442 (8)
C5—H5A	0.9500	C17—C18	1.389 (9)
C6—C7	1.516 (4)	C17—H17A	0.9500
C7—C8	1.546 (4)	C18—H18A	0.9500
C7—H7A	1.0000	C20—C21	1.395 (9)
C8—C9	1.507 (4)	C20—H20A	0.9500
C8—H8A	0.9900	C21—H21A	0.9500
C8—H8B	0.9900	C17X—C18X	1.394 (10)
C9—C16	1.467 (4)	C17X—H17B	0.9500
C10—C15	1.398 (4)	C18X—H18B	0.9500
C10—C11	1.406 (4)	C20X—C21X	1.389 (9)
C11—C12	1.388 (5)	C20X—H20B	0.9500
C11—H11A	0.9500	C21X—H21B	0.9500
			
N2—N1—C10	119.6 (2)	C12—C13—H13A	120.4
N2—N1—C7	113.1 (2)	C13—C14—C15	121.1 (3)
C10—N1—C7	125.1 (3)	C13—C14—H14A	119.5
C9—N2—N1	108.8 (2)	C15—C14—H14A	119.5
C2—C1—C6	120.8 (3)	C14—C15—C10	119.8 (3)
C2—C1—H1A	119.6	C14—C15—H15A	120.1
C6—C1—H1A	119.6	C10—C15—H15A	120.1
C3—C2—C1	118.9 (3)	C17X—C16—C21	110.9 (4)
C3—C2—H2A	120.5	C17—C16—C21	118.1 (5)
C1—C2—H2A	120.5	C17X—C16—C21X	119.5 (5)
C4—C3—C2	121.5 (3)	C17—C16—C21X	111.1 (4)
C4—C3—Br1	118.5 (2)	C17X—C16—C9	122.7 (4)
C2—C3—Br1	120.0 (2)	C17—C16—C9	123.9 (4)
C3—C4—C5	119.1 (3)	C21—C16—C9	117.9 (4)
C3—C4—H4A	120.5	C21X—C16—C9	117.8 (4)
C5—C4—H4A	120.5	C18—C19—F1	120.5 (5)
C4—C5—C6	120.9 (3)	C18X—C19—F1	120.2 (5)
C4—C5—H5A	119.6	C18—C19—C20	123.3 (5)
C6—C5—H5A	119.6	C18X—C19—C20	116.0 (5)
C5—C6—C1	118.8 (3)	F1—C19—C20	115.8 (4)
C5—C6—C7	120.6 (3)	C18—C19—C20X	115.7 (5)
C1—C6—C7	120.6 (3)	C18X—C19—C20X	122.6 (6)
N1—C7—C6	111.7 (2)	F1—C19—C20X	117.0 (4)
N1—C7—C8	101.2 (2)	C16—C17—C18	122.7 (6)
C6—C7—C8	114.3 (3)	C16—C17—H17A	118.7
N1—C7—H7A	109.8	C18—C17—H17A	118.7
C6—C7—H7A	109.8	C19—C18—C17	118.9 (7)
C8—C7—H7A	109.8	C19—C18—H18A	120.6
C9—C8—C7	102.8 (2)	C17—C18—H18A	120.6
C9—C8—H8A	111.2	C21—C20—C19	116.5 (6)
C7—C8—H8A	111.2	C21—C20—H20A	121.7
C9—C8—H8B	111.2	C19—C20—H20A	121.7
C7—C8—H8B	111.2	C20—C21—C16	119.9 (6)
H8A—C8—H8B	109.1	C20—C21—H21A	120.0
N2—C9—C16	122.8 (3)	C16—C21—H21A	120.0
N2—C9—C8	113.7 (3)	C16—C17X—C18X	122.6 (7)
C16—C9—C8	123.4 (3)	C16—C17X—H17B	118.7
N1—C10—C15	120.4 (3)	C18X—C17X—H17B	118.7
N1—C10—C11	120.4 (3)	C19—C18X—C17X	118.6 (8)
C15—C10—C11	119.2 (3)	C19—C18X—H18B	120.7
C12—C11—C10	119.7 (3)	C17X—C18X—H18B	120.7
C12—C11—H11A	120.2	C21X—C20X—C19	117.5 (6)
C10—C11—H11A	120.2	C21X—C20X—H20B	121.2
C13—C12—C11	121.1 (3)	C19—C20X—H20B	121.2
C13—C12—H12A	119.4	C20X—C21X—C16	118.6 (6)
C11—C12—H12A	119.4	C20X—C21X—H21B	120.7
C14—C13—C12	119.1 (3)	C16—C21X—H21B	120.7
C14—C13—H13A	120.4		
			
C10—N1—N2—C9	−168.5 (3)	C8—C9—C16—C17	−19.9 (5)
C7—N1—N2—C9	−4.5 (4)	N2—C9—C16—C21	−23.3 (5)
C6—C1—C2—C3	0.4 (5)	C8—C9—C16—C21	155.5 (4)
C1—C2—C3—C4	−2.2 (5)	N2—C9—C16—C21X	13.7 (5)
C1—C2—C3—Br1	177.1 (2)	C8—C9—C16—C21X	−167.4 (4)
C2—C3—C4—C5	1.4 (5)	C17X—C16—C17—C18	80.8 (12)
Br1—C3—C4—C5	−177.9 (2)	C21—C16—C17—C18	1.2 (4)
C3—C4—C5—C6	1.3 (5)	C21X—C16—C17—C18	−33.9 (4)
C4—C5—C6—C1	−3.0 (5)	C9—C16—C17—C18	176.7 (4)
C4—C5—C6—C7	176.6 (3)	C18X—C19—C18—C17	−85.6 (19)
C2—C1—C6—C5	2.2 (5)	F1—C19—C18—C17	178.7 (4)
C2—C1—C6—C7	−177.5 (3)	C20—C19—C18—C17	−9.2 (6)
N2—N1—C7—C6	128.1 (3)	C20X—C19—C18—C17	28.6 (6)
C10—N1—C7—C6	−69.1 (4)	C16—C17—C18—C19	5.1 (6)
N2—N1—C7—C8	6.1 (3)	C18—C19—C20—C21	6.8 (6)
C10—N1—C7—C8	168.9 (3)	C18X—C19—C20—C21	30.2 (7)
C5—C6—C7—N1	−42.2 (4)	F1—C19—C20—C21	179.2 (5)
C1—C6—C7—N1	137.4 (3)	C20X—C19—C20—C21	−80.2 (8)
C5—C6—C7—C8	71.9 (4)	C19—C20—C21—C16	−0.2 (7)
C1—C6—C7—C8	−108.4 (3)	C17X—C16—C21—C20	−30.3 (6)
N1—C7—C8—C9	−5.0 (3)	C17—C16—C21—C20	−3.5 (6)
C6—C7—C8—C9	−125.3 (3)	C21X—C16—C21—C20	82.0 (8)
N1—N2—C9—C16	179.7 (3)	C9—C16—C21—C20	−179.3 (4)
N1—N2—C9—C8	0.7 (4)	C17—C16—C17X—C18X	−77.7 (12)
C7—C8—C9—N2	3.0 (4)	C21—C16—C17X—C18X	34.0 (5)
C7—C8—C9—C16	−175.9 (3)	C21X—C16—C17X—C18X	−1.0 (4)
N2—N1—C10—C15	169.6 (3)	C9—C16—C17X—C18X	−178.8 (4)
C7—N1—C10—C15	7.7 (5)	C18—C19—C18X—C17X	87.1 (19)
N2—N1—C10—C11	−12.9 (4)	F1—C19—C18X—C17X	−175.8 (4)
C7—N1—C10—C11	−174.7 (3)	C20—C19—C18X—C17X	−28.3 (6)
N1—C10—C11—C12	−175.9 (3)	C20X—C19—C18X—C17X	9.7 (6)
C15—C10—C11—C12	1.7 (5)	C16—C17X—C18X—C19	−5.4 (6)
C10—C11—C12—C13	−0.7 (5)	C18—C19—C20X—C21X	−30.8 (6)
C11—C12—C13—C14	−0.5 (5)	C18X—C19—C20X—C21X	−7.4 (6)
C12—C13—C14—C15	0.6 (5)	F1—C19—C20X—C21X	177.9 (4)
C13—C14—C15—C10	0.5 (5)	C20—C19—C20X—C21X	81.3 (8)
N1—C10—C15—C14	176.0 (3)	C19—C20X—C21X—C16	0.8 (6)
C11—C10—C15—C14	−1.6 (5)	C17X—C16—C21X—C20X	3.1 (5)
N2—C9—C16—C17X	−168.4 (4)	C17—C16—C21X—C20X	29.6 (5)
C8—C9—C16—C17X	10.4 (5)	C21—C16—C21X—C20X	−79.9 (8)
N2—C9—C16—C17	161.2 (4)	C9—C16—C21X—C20X	−178.9 (4)

### Hydrogen-bond geometry (Å, º)

*Cg*1 is the centroid of the C10–C15 ring.

**Table:**

*D*—H···*A*	*D*—H	H···*A*	*D*···*A*	*D*—H···*A*
C7—H7*A*···*Cg*1^i^	1.00	2.60	3.522 (3)	153
C17—H17*A*···*Cg*1^ii^	0.95	2.99	3.752 (6)	138

Symmetry codes: (i) *x*, *y*+1, *z*; (ii) *x*, −*y*+1/2, *z*−3/2.

## References

[bb1] Bruker (2009). *SADABS*, *APEX2* and *SAINT* Bruker AXS Inc., Madison, Wisconsin, USA.

[bb2] Cosier, J. & Glazer, A. M. (1986). *J. Appl. Cryst.* **19**, 105–107.

[bb3] Fun, H.-K., Hemamalini, M., Samshuddin, S., Narayana, B. & Yathirajan, H. S. (2010). *Acta Cryst.* E**66**, o582–o583. 10.1107/S1600536810004435 PMC2983722 21580348

[bb4] Samshuddin, S., Narayana, B., Baktir, Z., Akkurt, M. & Yathirajan, H. S. (2011). *Der Pharma Chem.* **3**, 487–493.

[bb5] Samshuddin, S., Narayana, B., Yathirajan, H. S., Safwan, A. P. & Tiekink, E. R. T. (2010). *Acta Cryst.* E**66**, o1279–o1280. 10.1107/S1600536810015795 PMC2979444 21579379

[bb6] Sheldrick, G. M. (2008). *Acta Cryst.* A**64**, 112–122. 10.1107/S0108767307043930 18156677

[bb7] Spek, A. L. (2009). *Acta Cryst.* D**65**, 148–155. 10.1107/S090744490804362X PMC2631630 19171970

